# Thermographic and ultrasound assessment in patients with rheumatoid arthritis: can thermography detect subclinical synovitis at the wrist?

**DOI:** 10.1186/s41927-024-00435-1

**Published:** 2024-11-19

**Authors:** York Kiat Tan, Gek Hsiang Lim, Chin Chin Ooi, Voon Chee Ma, Bimal Mayur Kumar Vora

**Affiliations:** 1 Department of Rheumatology and Immunology, Singapore General Hospital, Outram Road, Singapore, 169608 Singapore; 2 Duke-NUS Medical School, Singapore, Singapore; 3 Yong Loo Lin School of Medicine, National University of Singapore, Singapore, Singapore; 4 Health Services Research Unit, Singapore General Hospital, Singapore, Singapore; 5 Radiography Department, Allied Health Division, Singapore General Hospital, Singapore, Singapore; 6 Health and Social Sciences Cluster, Singapore Institute of Technology, Singapore, Singapore; 7 Department of Diagnostic Radiology, Singapore General Hospital, Singapore, Singapore

**Keywords:** Thermography, Ultrasonography, Rheumatoid arthritis, Joints, Synovitis

## Abstract

**Background:**

Thermography is an emerging imaging modality which allows for a quick and objective measure of joint surface temperature in patients with rheumatoid arthritis (RA). To date, there are no published studies comparing thermography with ultrasonography in the subclinical assessment of joint inflammation at the wrist of patients with RA, and no published data on inter-rater reliability for multiple raters for thermographic assessment at the RA wrist. In our study comparing thermography and ultrasonography at the RA wrist, we aim to determine if thermography can detect subclinical synovitis. Additionally, we performed inter-reliability testing (multiple raters) for both thermography and ultrasonography.

**Methods:**

Thermographic (average (Tavg), maximum (Tmax) and minimum (Tmin) temperatures) and ultrasound (total grey-scale (TGS) score and total power Doppler (TPD) scores) parameters were compared between two patient groups: Asymptomatic Group (with non-swollen and non-tender wrists) and Symptomatic Group (with swollen and/or tender wrists). Among Asymptomatic Group patients, thermographic parameters were compared between those with and without wrist joint recess(es) having ultrasound synovitis (PD ≥ 1 or GS ≥ 2); Spearman’s correlation and simple linear regression were used to study the relationship between thermographic and ultrasound parameters. Intra-class correlation coefficient (ICC) was used for inter-rater reliability calculation.

**Results:**

Eighty-seven RA patients’ right wrists were imaged in this cross-sectional study. Thermographic temperatures, TPD and TGS scores were all significantly (*p* < 0.05) greater among Symptomatic Group versus Asymptomatic Group patients. Among Asymptomatic Group patients, thermographic temperatures were all significantly higher (*P* < 0.01) in wrists having joint recess(es) with ultrasound PD ≥ 1 or GS ≥ 2, while all thermographic parameters correlated significantly with TPD (correlation coefficient ranging from 0.43 to 0.48, *p* < 0.001) and TGS (correlation coefficient ranging from 0.33 to 0.37, *p* < 0.01). The ICC values based on a subset of images obtained for inter-reliability testing were high for thermography (0.994 to 0.998) and ultrasonography (0.933 to 0.952).

**Conclusions:**

Swollen and/or tender RA wrists displayed greater thermographic and ultrasound-detected joint inflammation. At clinically quiescent (non-swollen; non-tender) wrists, thermographic temperatures significantly correlated with ultrasound-detected joint inflammation.

**Clinical trial number:**

Not applicable.

## Background

Over the past two decades, there have been substantial growth in the rheumatoid arthritis (RA) literature [1] pertaining to the use of ultrasound and magnetic resonance imaging (MRI) as diagnostic, prognostic and outcome measurement tools in both trials and routine care settings [2, 3]. These modern imaging tools have the benefit of directly visualizing inflammatory and damage pathologies at the joints in patients with RA. They are more sensitive than conventional radiography (CR) in detecting bone erosions especially in early RA disease and are superior to clinical joint examination in the detection of joint/tendon inflammation [4]. Ultrasound power Doppler (PD) synovitis has been demonstrated to be a prognostic marker for RA flares and damage progression at the joints, while MRI bone marrow oedema (or osteitis) has been shown to be predictive of subsequent bone erosion formation [1]. About a decade ago, the European League Against Rheumatism (EULAR) provided a set of recommendations for the use of imaging in the clinical management of patients with RA [4] which span broadly across areas pertaining to RA diagnosis, prognosis and disease monitoring. For example, according to the EULAR recommendations [4], ultrasound and MRI can help improve the certainty of RA diagnosis above clinical criteria alone in cases of diagnostic doubt and help predict joint damage even in patients with RA who are in clinical remission. Nonetheless, ultrasound and MRI have their limitations as imaging tools [5, 6]. MRI scans are generally expensive and may not be feasible as a routine monitoring tool for RA patients. Ultrasound, on the other hand, may be subject to inter-observer reliability issues and can be time-consuming especially when used for scanning multiple different joint sites. Hence, there is a need to look at other affordable low-cost imaging technologies that may be feasible for use in the daily rheumatology practice setting. Infra-red thermal imaging (or thermography) is an emerging imaging tool which can be used for joint inflammation assessment by objectively quantifying joint surface temperatures in patients with RA [7]. Thermography is a relatively cheap, contactless and non-invasive imaging modality. With technological advancement, modern thermal cameras are compact, portable, and allows fast image acquisition [8]. They are therefore well-suited for use as adjunctive imaging tools in the Rheumatologist’s office. The wrist, essential for performing daily activities [9], is chosen for assessment in our study as it is commonly affected in patients with RA [10]. For example, among patients with early RA (within two years of diagnosis), more than half will have wrist pain and the vast majority (> 90%) will have wrist disease by 10 years [11]. Ultrasound is an established imaging modality for joint inflammation assessment [12] and can help detect subclinical synovitis in patients with RA [1, 4]. However, there is presently a lack of data on whether thermography may be similarly useful, especially in the detection of subclinical joint disease. In our study comparing thermography and ultrasonography at the wrist of RA patients, we aim to determine if thermography can help detect subclinical joint inflammation at the wrist. Additionally, we performed inter-reliability testing (multiple raters) for both thermography and ultrasonography.

## Methods

This single site cross-sectional study conducted at a local tertiary hospital included male or female patients from age 21 to 99 years old who fulfilled the 2010 RA classification criteria [13]. The patients were consecutively recruited at the hospital rheumatology outpatient clinics between December 2020 and July 2023. Female pregnant patient(s) were excluded from the study. This study conforms to the relevant research ethnical guidelines and was approved by SingHealth Centralised Institutional Review Board (CIRB) (2020/2669). All patients provided their informed consent before they were recruited into the study.

### Physical examination

The physical examination and imaging (thermal and ultrasound) assessments were carried out during the same study visit. Clinical joint assessments were performed by trained rheumatology nurses (who received prior standardized training) blinded to the findings from thermal and ultrasound imaging. Joint swelling and tenderness were elicited as either present (Yes = 1) or absent (No = 0). For standardization, the RA patients were categorized into the following two patients groups based on their clinical swelling and tenderness status at the right wrist: (1) Asymptomatic Group patients with non-swollen and non-tender wrists and (2) Symptomatic Group patients with swollen and/or tender wrists.

### Imaging assessment at the wrist

For ultrasonography, standardized imaging was carried out following the EULAR guidelines [14] by a rheumatologist who was experienced in musculoskeletal ultrasound imaging while being blinded to the outcomes from the thermal imaging, whereas a separate study personnel carried out the thermography while being blinded to the outcomes from the ultrasound imaging. The Mindray M9 ultrasound machine (Mindray Bio-Medical Electronics Co., Ltd., Shenzhen, China) with a L14-6Ns linear probe was used for ultrasound scanning with the following settings: Doppler frequency 5.7 MHz; pulse repetition frequency (PRF) of 700 Hz. Ultrasound grey-scale (GS) synovial hypertrophy and PD were graded semi-quantitatively (0 being normal; 1 being mild; 2 being moderate and 3 being severe) based on previously published validated scoring methods [15, 16]. The dorsal wrist was scanned at the (i) distal radio-ulnar and (ii) radio-carpal/inter-carpal recesses and the GS and PD sub-scores at these recesses were summed to derive the total grey-scale (TGS) score and total power Doppler (TPD) scores, respectively. For example, for TGS score, if the GS sub-scores at the (i) distal radio-ulnar and (ii) radio-carpal/inter-carpal recesses were “1” and “2” respectively, the TGS score will be “3” (i.e. sum of “1” and “2”). For TPD score, if the PD sub-scores at the (i) distal radio-ulnar and (ii) radio-carpal/inter-carpal recesses were “0” and “1” respectively, the TPD score will be “1” (i.e. sum of “0” and “1”).

The dorsal wrist was scanned at two joint recesses separately (i.e. the (i) distal radio-ulnar and (ii) radio-carpal/inter-carpal recesses) for a more representative picture of ultrasound PD and GS joint inflammation at the entire wrist (instead of a more limited scanning at the wrist) to allow for comparison with thermography of the wrist. Additionally, ultrasound synovitis at a wrist joint recess is defined as having an ultrasound score of either PD ≥ 1 or GS ≥ 2 [17–19].

For thermographic assessment, standardized imaging based on previously described established methods [8, 20, 21] was carried out in a windowless (draft-free) room with an ambient temperature of around 23 °C [21]. The dorsal view of the hand was imaged using a high performance portable thermal camera FLIR T640 (FLIR Systems AB, Sweden) with predefined emissivity value of 0.98 for skin [8]. The settings of the thermal camera were as follows: pixel resolution 640 × 480; thermal sensitivity of < 30 milli-Kelvin (mK) at 30 °C. As per usual practice, patients were rested 15 min prior to thermal imaging to allow for acclimatisation [21]. Objects obscuring the view of the thermal camera (e.g. watches, jewelleries) were removed. The patient’s hand was placed in a neutral position on a flat table top and the thermogram acquired with the thermal camera positioned 50 cm above the hand. The region of interest (ROI) [8, 22] on the thermogram was segmented manually by placing a rectangular box over the wrist area. From the wrist ROI, the following thermographic parameters were recorded for subsequently analysis: average (Tavg), maximum (Tmax) and minimum (Tmin) temperatures.

### Reliability analysis at the wrist

From the 87 RA patients, a subset of 30 anonymized static images were obtained for inter-rater analysis (for ultrasound: 10 images each for PD and GS joint inflammation; for thermography: 10 images to obtain Tavg, Tmax and Tmin). 4 different raters (a rheumatologist experienced with ultrasonography; a musculoskeletal radiologist and two ultrasonographers) were involved in this reliability exercise (with their assessments performed while being blinded to the clinical details). The 4 raters underwent a standardization session prior to this reliability exercise. For ultrasonography, images representing a wide range of different grades of GS and PD joint inflammation at the wrist were obtained for inter-rater reliability analysis [15]. For thermography, 10 thermograms in patients with varying wrist sizes were selected for inter-rater reliability analysis.

### Statistical analysis

Thermographic parameters (Tavg, Tmax and Tmin) and ultrasound parameters (TGS and TPD scores) were compared between patients with non-swollen and non-tender wrists (Asymptomatic Group) and patients with swollen and/or tender wrists (Symptomatic Group) using a 2-independent samples t-test. Among Asymptomatic Group patients (with non-swollen and non-tender wrists), thermographic parameters were also compared between RA patients with and without wrist joint recess(es) having ultrasound synovitis (PD ≥ 1 or GS ≥ 2) [17–19]; Spearman’s correlation and simple linear regression were used to study the relationship between thermographic and ultrasound parameters. The statistical significance level was set at *p* < 0.05. Intra-class correlation coefficient (ICC) was used to assess the inter-rater reliability among the 4 raters in their scoring of ultrasound GS and PD joint inflammation and manual segmentation of the wrist ROI on the thermograms (to obtain Tavg, Tmax and Tmin). The interpretation of ICC values are as follows: low (less than 0.40); moderate (between 0.40 and 0.75); substantial (between 0.75 and 0.90); excellent (greater than 0.90) [23]. Statistical analyses were carried out using Stata 17 (StataCorp. 2021. Stata Statistical Software: Release 17. College Station, TX: StataCorp LLC).

## Results

### Baseline patients’ characteristics

A total of 87 right wrist were evaluated from 87 RA patients. At baseline, the mean (SD) age of the patient, disease duration and 28-joint disease activity score (DAS28) were 58.2 (12.5) years, 27.8 (48.2) months and 3.64 (1.21), respectively. 69 out of 87 patients (79.3%) were female while 64 out of 87 patients were Chinese (73.6%). 56 out of 87 patients (64.4%) were on oral prednisolone (among those on prednisolone, the mean (SD) of the prednisolone dose was 4.6 (2.1) mg per day). All patients were on one or more of the following disease-modifying anti-rheumatic drugs (DMARDs): leflunomide, methothrexate, sulfasalazine and/or hydroxychloroquine. Out of the 84 right wrist distal radio-ulnar joint recesses scanned, 40 had ultrasound score of PD ≥ 1 (40 out of 84, 47.6%) and 20 had ultrasound score of GS ≥ 2 (20 out of 84, 23.8%). Out of the 84 right wrist radio-carpal/inter-carpal recesses scanned, 58 had ultrasound score of PD ≥ 1 (58 out of 84, 69.0%) and 32 had ultrasound score of GS ≥ 2 (32 out of 84, 38.1%).

### Comparison between patients from asymptomatic group and symptomatic group

For thermography, all thermographic temperatures (Tavg, Tmax and Tmin) were statistically significantly (all p-values < 0.05) greater (see Table 1) among Symptomatic Group (those with swollen and/or tender wrists) patients versus Asymptomatic Group (those with non-swollen and non-tender wrists) patients (mean (SD) Tavg were 32.0 (1.6) versus 31.0 (1.5) respectively, *P* = 0.006; mean (SD) Tmax were 33.0 (1.7) versus 32.0 (1.5) respectively, *p* = 0.003; mean (SD) Tmin were 30.7 (1.6) versus 29.9 (1.5) respectively, *p* = 0.03). For ultrasonography, the TPD and TGS scores were both statistically significantly (all p-values < 0.001) greater (see Table 1) among Symptomatic Group patients versus Asymptomatic Group patients (mean (SD) TPD score were 2.4 (1.4) versus 1.0 (1.2) respectively, *p* < 0.001; mean (SD) TGS were 3.1 (1.3) versus 2.1 (1.0) respectively, *p* = 0.0002).

**Table 1:** Comparing imaging findings between patient groups

Imaging parameter	Asymptomatic group patients with non-swollen and non-tender wrists (*n* = 50)	Symptomatic group patients with swollen and/or tender wrists (*n* = 37)	*p*-value
TPD score, mean(SD)	1.0 (1.2)	2.4 (1.4)	< 0.001***
TGS score, mean(SD)	2.1 (1.0)	3.1 (1.3)	0.0002***
Tavg, mean(SD)	31.0 (1.5)	32.0 (1.6)	0.006**
Tmax, mean(SD)	32.0 (1.5)	33.0 (1.7)	0.003**
Tmin, mean(SD)	29.9 (1.5)	30.7 (1.6)	0.03*

Abbreviations TPD, total power Doppler; TGS, total grey-scale; Tavg, average temperature

Tmax, maximum temperature; Tmin, minimum temperature

Statistically significant: ***p < 0.001, **p < 0.01, *p < 0.05

### Comparison of thermal and ultrasound imaging among patients in asymptomatic group

**Fig. 1:**
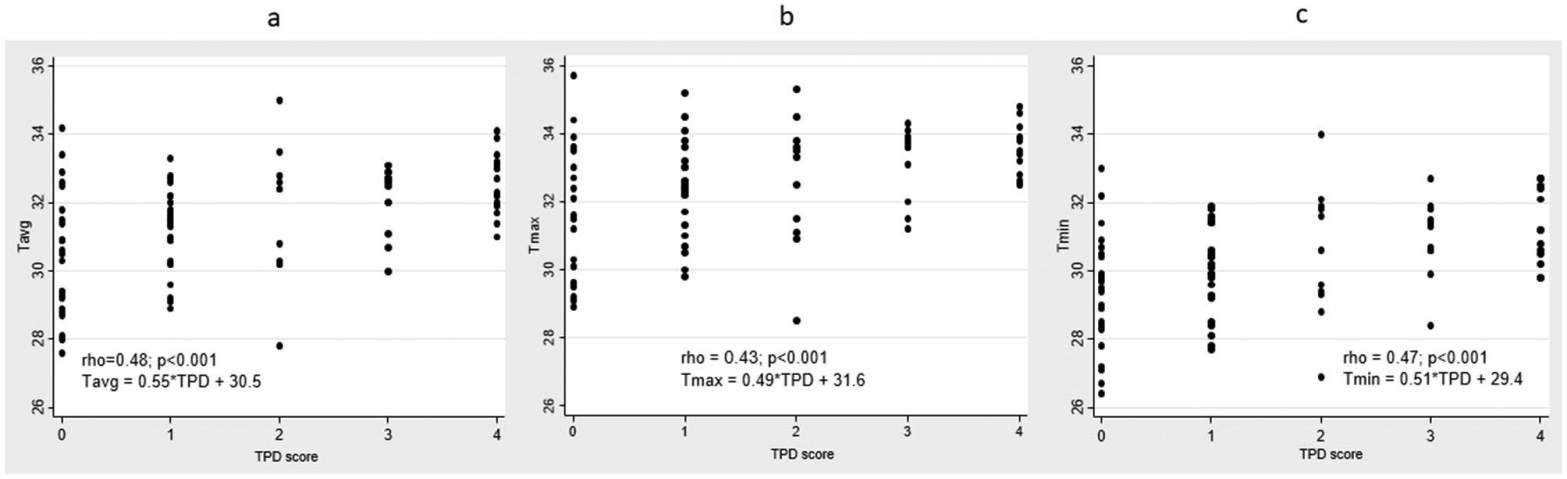
Thermographic parameters versus TPD scores. **a**, Tavg versus TPD scores. **b**, Tmax versus TPD scores. **c**, Tmin versus TPD scores. Abbreviations: Tavg, average temperature; Tmax, maximum temperature; Tmin, minimum temperature; TPD, total power Doppler

**Fig. 2:**
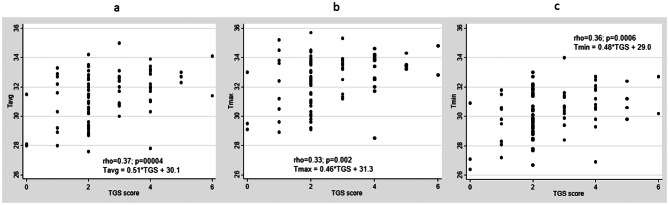
Thermographic parameters versus TGS scores. **a**, Tavg versus TGS scores. **b**, Tmax versus TGS scores. **c**, Tmin versus TGS scores. Abbreviations: Tavg, average temperature; Tmax, maximum temperature; Tmin, minimum temperature; TGS, total grey-scale

Among Asymptomatic Group patients (with non-swollen and non-tender wrists), thermographic temperatures (Tavg, Tmax and Tmin) were all significantly higher (see Table 2) comparing patients with and without ultrasound synovitis (PD ≥ 1 or GS ≥ 2) at the joint recess(es) of the wrist (mean (SD) Tavg were 31.5 (1.2) versus 30.3 (1.7), respectively, *P* = 0.007); mean (SD) Tmax were 32.5 (1.2) versus 31.3 (1.7), respectively, *p* = 0.007; mean (SD) Tmin were 30.4 (1.3) versus 29.3 (1.5), respectively, *p* = 0.008). Furthermore, all thermographic parameters (Tavg, Tmax and Tmin) correlated significantly with TPD score (Tavg, rho = 0.48, *p* < 0.001; Tmax, rho = 0.43, *p* < 0.001; Tmin, rho = 0.47, *p* < 0.001) and TGS score (Tavg, rho = 0.37, *p* = 0.0004; Tmax, rho = 0.33, *p* = 0.002; Tmin, rho = 0.36, *p* = 0.0006). For TPD score, linear regression analysis (see Fig. 1) revealed that with an increase by 1 in TPD score, Tavg, Tmax and Tmin would increase by 0.55, 0.49 and 0.51 respectively. For TGS score, linear regression analysis (see Fig. 2) revealed that with an increase by 1 in TGS score, Tavg, Tmax and Tmin would increase by 0.51, 0.46 and 0.48, respectively.

**Table 2:** Comparing thermographic temperatures within asymptomatic group patients

Thermographic parameter	Patients without PD ≥ 1 or GS ≥ 2 at the wrist joint recess(es) (*n* = 21)	Patients with PD ≥ 1 or GS ≥ 2 at one or more wrist joint recess(es) (*n* = 29)	*p*-value
Tavg, mean(SD)	30.3 (1.7)	31.5 (1.2)	0.007**
Tmax, mean(SD)	31.3 (1.7)	32.5 (1.2)	0.007**
Tmin, mean(SD)	29.3 (1.5)	30.4 (1.3)	0.008**

Abbreviations PD, power Doppler; GS, grey-scale; Tavg, average temperature; Tmax, maximum temperature; Tmin, minimum temperature. Statistically significant: **p < 0.01

### Inter-rater reliability analysis

The ICC results for both thermal and ultrasound imaging based on the subset of images used for inter-rater reliability analysis are summarized in Table 3. The ICC values for the 4 raters for all thermographic parameters (Tavg, Tmax and Tmin) were high and ranged from 0.994 to 0.998 (Tavg: ICC 0.998, 95% CI 0.995 to 0.999; Tmax: ICC 0.996, 95% CI 0.990 to 0.999; Tmin, ICC 0.994, 95% CI 0.985 to 0.998). The ICC values for the 4 raters were also high for ultrasound imaging (both PD and GS joint inflammation) and ranged from 0.933 to 0.952 (PD joint inflammation: ICC 0.952, 95% CI 0.882 to 0.986; GS joint inflammation: ICC 0.933, 95% CI 0.837 to 0.981).

**Table 3:** Inter-rater reliability analysis

Imaging parameter	Number of raters	Intra-class correlation coefficient (95% CI)
PD joint inflammation	4	0.952 (0.882, 0.986)
GS joint inflammation	4	0.933 (0.837, 0.981)
Tavg	4	0.998 (0.995, 0.999)
Tmax	4	0.996 (0.990, 0.999)
Tmin	4	0.994 (0.985, 0.998)

Abbreviations PD, power Doppler; GS, grey-scale

Tavg, average temperature; Tmax, maximum temperature; Tmin, minimum temperature

## Discussion

There has been an increase in interest in the use of thermography in the past decade in the assessment of inflammatory and degenerative joint conditions based on publication trends [24, 25]. In our study, we have demonstrated that thermographic temperatures and ultrasound joint inflammation scores were significantly greater in RA patients with swollen and/or tender wrist when compared to those with clinically quiescent wrist (non-swollen and non-tender). Swollen and tender joint counts are routinely performed in the rheumatology clinic as part of patient disease activity assessment and are components of the widely utilized composite disease activity score DAS28 [26] and the American College of Rheumatology (ACR) response criteria [27]. Interestingly, a recent small-scale study [20] evaluated the use of a novel combined thermography and clinical joint assessment (CTCA) approach (e.g. by increasing the clinical assessment weightage of joint swelling and tenderness to a composite score derived from both thermography and clinical joint assessment) and found that the CTCA approach can help discriminates ultrasound-detected joint inflammation severity in RA at more joint sites compared to thermography alone. While promising, the authors concluded that more validation work using this novel approach will be required in larger RA cohorts [20]. For the first time, our study demonstrated a significant correlation between thermographic findings and ultrasound joint inflammation outcomes at clinically quiescent (non-swollen and non-tender) RA wrists. In an imaging study by Ogishima et al. [28] using CR, ultrasonography and low field MRI to examine the hand joints of 77 patients with RA, subclinical synovitis (detected based on ultrasound and low field MRI criteria in the absence of joint swelling and tenderness) of the small joints of the hand showed radiologic progression to bone destruction [28]. In a separate ultrasound imaging RA study (*n* = 59) by Inamo et al. [29] involving the ankles and feet, it was observed that physician visual analog scale (VAS) scores were significantly reduced in patients with ultrasound-detected subclinical synovitis (while patient and pain VAS scores were significantly reduced only among patients with no synovitis) indicating subclinical synovitis was often unnoticed by physicians. Importantly, the study [29] also demonstrated that ultrasound-detected subclinical synovitis substantially impairs patients’ quality of life (QoL) and functional ability. A small-scale study by Gent et al. [30] including RA patients without clinical disease (*n* = 29), positron emission tomography (PET) scanning at the hand and wrist joints revealed the presence of subclinical synovitis (with enhanced uptake) in over half of the patients, with the highest cumulative PET scores related to disease flare within 6 months. The above 3 studies [28–30] suggest that subclinical synovitis detected at the joint level in RA patients can led to undesirable outcomes (including radiologic progression in the study by Ogishima et al. [28], worsened QoL/functional ability in the study by Inamo et al. [29] and disease flare in the study by Gent et al. [30]). At present, it is not known whether subclinical inflammation detected by thermography can lead to similar undesirable outcomes and well-designed prospective longitudinal RA studies would be required to answer this question.

The use of standardized consensus-based definition of ultrasound joint inflammation has helped improved the reliability of ultrasound as an assessment tool in RA [31]. Following the published validated EULAR-Outcome Measures in Rheumatology (EULAR-OMERACT) ultrasound scoring methods [15, 16], we have obtained high inter-rater reliability based on our ICC results. In comparison, there has been much less validation work on reliability testing pertaining to the use of thermography for joint inflammation assessment in RA. To the best of our knowledge, our present study is the first to report on the inter-rater reliability for multiple raters with regard to thermographic assessment at the wrist of patient with RA. Like our study, Ahn et al. [32] has shown an excellent inter-observer consistency (ICC greater than 0.9) for thermographic assessment at the knee among 30 subjects with knee arthritis (of which 12 patients had RA). Therefore, further thermographic studies looking at reliability testing will be required in patients with RA, and these should ideally include assessment at various different joint sites.

Previous studies published on the use of thermography in RA had compared thermal imaging with the use of ultrasound [8, 33–35]. In a small cohort study (*n* = 37 RA patients) [33] comparing thermography with ultrasonography at the joint sites of the bilateral hands (including wrists), it was demonstrated that joints displayed significantly higher thermographic temperatures when ultrasound PD joint inflammation and GS joint inflammation were present. In a separate knee study by Vasdev et al. [34] including 50 RA patients and 50 control subjects, ultrasound-detected PD ultrasound findings had significant correlation with knee-thigh temperature differential. In another study by Gizi´nska et al. [35] (including 81 RA subjects and 39 healthy controls) examining the RA foot, there were no significant differences in the average temperatures in joints with or without inflammation detected on ultrasound (except in the first metatarsophalangeal joint (MTPJ) of the right foot and second MTPJ of the left foot). Taken together, the above three studies [33–35] and our present study suggest that the usefulness of thermography in RA may differ according to different joint sites. Hence, future larger scale well-designed RA studies comparing the usefulness of thermography at various joint sites will be warranted.

Our study has its limitations. This is a cross-sectional study with both thermal and ultrasound imaging performed at a single time-point at the wrist of patients with RA on conventional DMARDs. Moreover, we only performed joint inflammation assessment without evaluating for structural joint damage. Therefore, future prospective longitudinal RA studies with imaging performed at multiple time-points will be required with comparative analysis performed between thermography and ultrasonography at various different joint sites and in RA patients with different clinical profile (e.g. those on conventional DMARDs versus those on biological DMARDs treatment, etc.). These studies should ideally include measurements of both joint inflammation and damage outcomes. In this study, we did not look for concomitant osteoarthritis (OA) at the wrist. Future well designed studies will need to look specifically at whether concomitant OA (whether primary or secondary to RA) can influence the imaging outcomes at the RA wrist. As joint pain without swelling can be due to other reasons, another limitation of the study is the lack of a disease-control group for comparison. Future studies on thermal imaging in patients with RA should ideally include appropriate disease-control group for comparison.

## Conclusions

In summary, we have demonstrated that swollen and/or tender wrists have greater thermographic temperatures and ultrasound-detect joint inflammation when compared to clinically quiescent (non-swollen and non-tender) wrists. For the first time, we have demonstrated a significant correlation between thermographic temperatures and ultrasound-detected joint inflammation outcomes at clinically quiescent (non-swollen and non-tender) RA wrists. The high inter-rater reliability demonstrated for both thermography and ultrasonography further strengthens the validity of our results. Our study has also generated more research questions on the utility of thermal imaging in RA, for example whether thermography can be useful as a monitoring tool for residual joint disease detection after initial improvement of joint symptoms and whether it can help guide subsequent treatment (e.g. steroids and/or DMARDs) tapering. Other scenarios would be whether subclinical joint inflammation detected by thermography can help RA disease prognostication, such as predicting disease flare and/or damage progression. Thermography appears promising as a joint inflammation assessment tool in RA and answering these questions would be an important research agenda for the application of thermal imaging in RA.

## Data Availability

Data are available from the corresponding author on reasonable request.

## Abbreviations

CI: Confidence interval
CIRB: Centralised Institutional Review Board
CR: Conventional radiography
DAS28: 28-joint disease activity score
DMARDs: Disease-modifying anti-rheumatic drugs
EULAR: European League Against Rheumatism
GS: Grey-scale
ICC: Intra-class correlation coefficient
MRI: Magnetic resonance imaging
MTPJ: Metatarsophalangeal joint
OMERACT: Outcome Measures in Rheumatology
OA: Osteoarthritis
PD: Power Doppler
PET: Positron emission tomography
PRF: Pulse repetition frequency
RA: Rheumatoid arthritis
SD: Standard deviation
Tavg: Average temperature
TGS: Total grey-scale
Tmax: Maximum temperature
Tmin: Minimum temperature
TPD: Total power Doppler
